# Honey-Stabilized Alginate Nanoparticles Derived from Sargassum: Synthesis, Physicochemical Characterization and Colloidal Stability

**DOI:** 10.3390/polym18080996

**Published:** 2026-04-20

**Authors:** Hannia A. Ramírez-Lara, Ashley J. Gutierrez-Onofre, René Salgado-Delgado, Areli Marlén Salgado-Delgado, Iliana C. Martínez-Ortíz, Nahomi Y. Degollado-Hernández, Igor Garcia-Atutxa, Francisca Villanueva-Flores

**Affiliations:** 1 Centro de Investigación en Ciencia Aplicada y Tecnología Avanzada (CICATA) Unidad Morelos del Instituto Politécnico Nacional (IPN), Xochitepec C.P. 62790, Morelos, Mexico; 2 Tecnológico Nacional de México, Instituto Tecnológico (IT) de Zacatepec, Calzada Tecnológico 27, Centro, Zacatepec C.P. 62780, Morelos, Mexico; 3 División Académica de Mecánica Industrial, Universidad Tecnológica Emiliano Zapata del Estado de Morelos, Av. Universidad Tecnológica 1, Palo Escrito, Emiliano Zapata C.P. 62765, Morelos, Mexico; 4 Escuela Politécnica Superior, Universidad Católica de Murcia (UCAM), Av. de los Jerónimos, C.P. 30107 Murcia, Spain

**Keywords:** *Sargassum fluitans*, alginate, nanoparticles, α-L-guluronic acid, β-D-mannuronic acid

## Abstract

Massive pelagic *Sargassum* influxes along Caribbean coasts have created an urgent need for valorization routes for this biomass. Here, sodium alginate was extracted from *Sargassum fluitans* collected at Chuburná Beach, Yucatán, Mexico, using a multistep extraction involving 0.2% formaldehyde pretreatment at 4 °C and brief heating at 65–70 °C, and subsequently used to prepare calcium-crosslinked alginate nanoparticles by ionotropic gelation. To our knowledge, this is the first direct synthesis of alginate nanoparticles from non-commercial alginate extracted from pelagic *S. fluitans*. An extraction yield of 18.7 ± 0.05% (mean ± SD, *n* = 3) was obtained, and UV–Vis, FTIR, and NMR analyses confirmed the characteristic structural features of alginate. ^1^H NMR revealed an M-rich composition (F_M = 0.61, F_G = 0.39; M/G = 1.54) with short guluronate blocks (N_G>1 = 2.42), whereas ^13^C NMR corroborated the presence of both β-D-mannuronic and α-L-guluronic acid residues. SEM images showed predominantly spherical-to-subspherical nanoparticles with representative dry diameters of 233–269 nm, whereas DLS measurements at 0, 24, and 72 h revealed a dominant volume-based nanoscale population with main peaks at 12.75–15.31 nm and PDI values of 0.229–0.291, indicating reasonable short-term colloidal stability at room temperature. These results demonstrate that pelagic *S. fluitans* can serve as a viable feedstock for the production of structurally preserved alginate and calcium-crosslinked alginate nanoparticles. The study supports converting recurrent *Sargassum* biomass into higher-value polysaccharide-based materials and provides a basis for future application-specific evaluation of these nanomaterials.

## 1. Introduction

Sargassum is a brown macroalga that has recently emerged as a significant global environmental problem, especially along the Caribbean and Mexican coasts. Since approximately 2011, unprecedented blooms of pelagic Sargassum in the tropical Atlantic have led to massive beach inundations, resulting in significant ecological, economic, and social impacts [1]. In affected regions, thick mats of Sargassum wash ashore and decompose, darkening coastal waters, killing seagrasses and corals, and disrupting local fisheries and tourism [2,3]. Segaran et al. (2023) noted that Sargassum inundations over the past decade have become a new norm, linked to oceanographic changes, and thus, demand scientific attention to their multifaceted impacts [1].

One primary concern is the effect of decaying Sargassum on human health and coastal ecosystems. As the seaweed piles rot on beaches, they release noxious gases, chief among them hydrogen sulfide (H_2_S), which emits a strong “rotten egg” odor and poses health hazards. In the marine environment, the impacts are likewise severe. Massive Sargassum “brown tides” in the Mexican Caribbean resulted in reduced water quality and oxygen levels (hypoxia/anoxia), leading to eutrophication and the die-off of important biota [3]. Nearshore seagrass meadows suffered up to ~99% biomass loss, and corals experienced extensive mortality under these low-oxygen conditions [4,5].

Therefore, the influx of Sargassum has motivated research into the utilization and valorization of its biomass. A promising avenue is the extraction of alginate from Sargassum as a value-added product. Alginate is a polysaccharide found in the cell walls of brown algae (including Sargassum) and is already widely used as a natural biopolymer. Mohammed et al. (2020) demonstrated that Sargassum can serve as an effective feedstock for alginate production, although traditional processes yield low quantities of variable quality [6]. Typically, commercial alginates (e.g., from kelps like *Macrocystis* or *Laminaria*) comprise up to ~40% of the seaweed’s dry weight [6]. Sargassum species also contain appreciable amounts of alginate. However, they have historically been less favored due to lower yields (<20% dry weight) and higher impurity levels from conventional extraction. Nevertheless, given the “freely available and abundant biomass” of Sargassum in the Caribbean, there is strong interest in converting this environmental challenge into a source of high-value alginate [7]. These same properties also make alginate an excellent matrix for forming nanoparticles and other advanced materials for drug delivery and food packaging, due to its biocompatibility, biodegradability, and ability to encapsulate therapeutic molecules, thereby protecting them and enabling controlled release [8,9,10]. In summary, transforming Sargassum into alginate products, such as alginate nanoparticles for drug delivery, is a compelling strategy that adds value to the biomass and addresses a pressing waste problem [11,12].

Conventional alginate extraction from brown seaweeds (like Sargassum) relies on long-established steps (dating back to early 20th-century methods) and has proven effective for isolating alginic acid. Typically, it involves sequential treatment with dilute formaldehyde (to remove pigments and phenolic compounds, thereby preserving alginate quality), followed by hot alkali and acid stages, and finally precipitation with calcium chloride or ethanol to recover the alginate [13]. These time-tested methods have enabled the production of alginate at scale and a usable product, but they also come with some trade-offs. For instance, prolonged and/or harsh thermal exposure during extraction, particularly when combined with strongly alkaline conditions, can contribute to alginate depolymerization, lowering the polymer’s molecular weight and viscosity [14]. Additionally, the process requires significant quantities of chemicals (strong acids, bases, and additives such as formaldehyde), thereby increasing operational costs and necessitating careful waste handling [13]. Overall, while conventional extraction is robust and effective, there is growing interest in refining it to be gentler and more sustainable, for example, by reducing harsh conditions and chemical inputs, so that high-quality alginate can be obtained more efficiently and with a smaller environmental footprint. However, the present study did not aim to develop a formaldehyde-free extraction route; rather, it applied a literature-based protocol in which formaldehyde was retained as a pretreatment step. For pelagic *Sargassum*, however, the large quantity of biomass available during bloom events does not necessarily imply consistent quality. Once the material washes ashore and undergoes prolonged decay, microbial decomposition, oxidation, and accumulation of sand, salts, and other impurities may compromise alginate quality and limit its suitability for higher-value applications. Accordingly, valorization strategies are likely to be more effective when the biomass is collected before advanced beach-cast deterioration or is processed rapidly after collection.

In this study, alginate was extracted from *Sargassum fluitans* collected along the coast of Chuburná Beach, Yucatán, Mexico. The present work should therefore be interpreted as a proof-of-concept for pelagic biomass collected before severe post-stranding deterioration, rather than as direct evidence that heavily decomposed stranded material would perform equivalently. Any translation toward food, feed, or biomedical end uses would require controlled feedstock collection before advanced decay, rapid preprocessing, and application-specific quality control. *S. fluitans* exhibits specific characteristics that set it apart within the genus *Sargassum*. This alga is holopelagic, meaning it spends its entire life floating freely in the ocean, without a benthic phase attached to a substrate [15]. Here, we outline a two-step approach: first, the extraction of alginate from pelagic *S. fluitans* using a literature-based multistep procedure conducted under controlled conditions; second, the fabrication of alginate-based nanoparticles for potential biomedical applications. The purpose was not to introduce a fundamentally new extraction chemistry, but to evaluate whether pelagic *S. fluitans* from this region could yield structurally preserved alginate suitable for direct nanoparticle synthesis. In practical terms, this type of application is expected to depend on feedstock selection and prompt preprocessing to reduce the adverse effects of post-collection degradation. We employed a controlled extraction procedure intended to preserve alginate quality, rather than claiming a substantially lower-temperature or lower-chemical process than those already used in the field. Accordingly, this work should not be interpreted as a formaldehyde-free or reduced-chemical extraction approach. After extracting the alginate, we used it as a matrix to form polymeric nanoparticles via ionic crosslinking. In this method, alginate macromolecules were cross-linked with calcium ions under aqueous conditions, thereby inducing nanoparticle formation without conditions expected to compromise the polymer’s structure. This controlled crosslinking step ensured that the resulting alginate nanoparticles retained their structural integrity and functional groups, which is crucial for downstream bio-applications. We then characterized the alginate and the nanoparticles with a suite of analytical techniques: Ultraviolet–Visible (UV–Vis) spectroscopy (Thermo Scientific, Evolution One Plus UV-Vis spectrophotometer, Waltham, MA, USA)to verify any distinct absorbance features or confirm successful drug loading (if applicable), Fourier-transform infrared spectroscopy (FTIR) to identify characteristic functional groups and confirm alginate’s chemical signature, scanning electron microscopy (SEM) (JEOL, JSM-5600LV scanning, electron microscope, Tokyo, Japan) to visualize nanoparticle morphology and size. These characterization results confirmed that we obtained nanoscale alginate particles with the desired size (in the nanometer range) and good colloidal stability. The spectra and microscopic images indicated that the alginate’s key functional groups (e.g., carboxylate groups) were intact. Overall, *S. fluitans*-derived alginate nanoparticles exhibit properties that hold promise for advanced biotechnological and biomedical applications, although any such use would require rigorous physicochemical, contaminant, and batch-to-batch quality evaluation. In the following sections, we discuss in detail the extraction protocol, nanoparticle synthesis, and characterization, highlighting the suitability of pelagic Sargassum-derived alginate as a feedstock for structurally characterized nanoparticle formulations.

## 2. Materials and Methods

**Figure 1:**
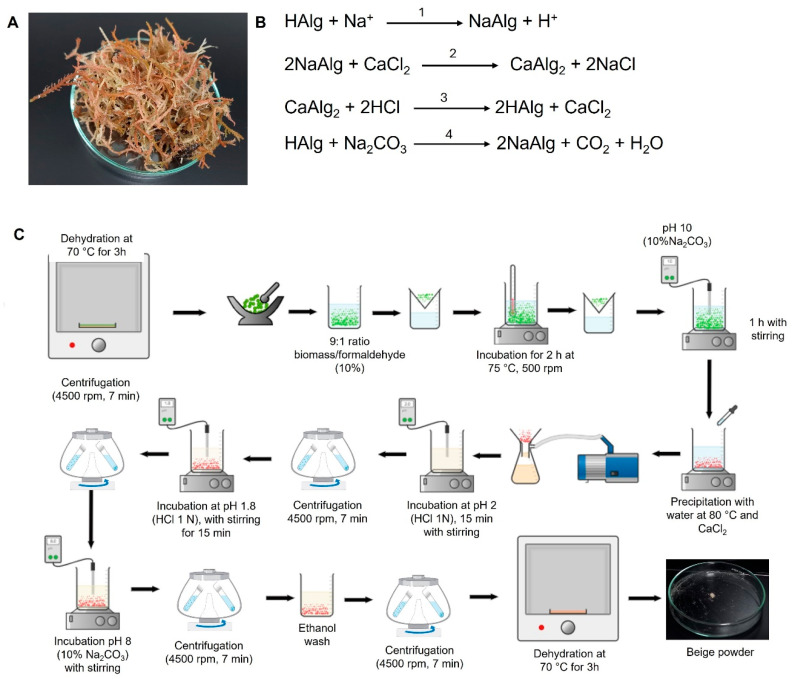
Extraction of sodium alginate from *S. fluitans*. (**A**) Fresh *S. fluitans* collected from Chuburná Beach, Yucatán, Mexico. (**B**) Chemical steps involved in the extraction of alginate from *S. fluitans*: (1) Alginic acid (HAlg) reacts with sodium ions (Na^+^) to yield sodium alginate (NaAlg) and release hydrogen ions (H^+^). (2) Sodium alginate subsequently reacts with calcium chloride (CaCl_2_) to form calcium alginate (CaAlg_2_) and sodium chloride (NaCl). (3) Calcium alginate then reacts with hydrochloric acid (HCl) to regenerate alginic acid (HAlg) and calcium chloride (CaCl_2_). (4) Finally, alginic acid reacts with sodium carbonate (Na_2_CO_3_) to produce sodium alginate (NaAlg), carbon dioxide (CO_2_), and water. (**C**) Experimental workflow for the extraction of alginate from *S. fluitans*.

Samples of *S. fluitans* were collected at Chuburná Beach, Yucatán, Mexico, in June 2023 and were kindly provided by Gustavo Chi-Dzul (Figure 1A). The algae were collected from the sea surface, thoroughly rinsed with deionized water, and frozen at −20 °C until use.

### 2.1. Purification of Alginate from Sargassum

The extraction of sodium alginate from *Sargassum* was carried out following a literature-based procedure adapted from previously reported methodologies [16,17], as detailed below. The operative parameters used in this study were selected based on those reports and applied here to pelagic *S. fluitans* under controlled conditions; thus, the procedure should be interpreted as literature-derived rather than as a de novo extraction method.

To remove initial contaminants, the Sargassum was thoroughly washed with deionized water and cut into smaller pieces with scissors to facilitate drying. Samples were dehydrated in a forced-convection oven (Yamato Scientific, model DKN602C, Tokyo, Japan) at 60 °C for 12 h, or until a constant weight was reached, as a controlled dehydration step to avoid more aggressive thermal exposure prior to extraction. Subsequently, the dried samples were frozen with liquid nitrogen and ground in a porcelain mortar to obtain a fine brown powder intended for subsequent extraction.

To remove phenolic compounds and pigments that may degrade or contaminate alginate, the powder was immersed in a 0.2% formaldehyde solution (Merck, F8775, Darmstadt, Germany) for 18 h at 4 °C under gentle agitation at 200 rpm, using a 9:1 solution-to-sample mass ratio. After formaldehyde pretreatment, the biomass was vacuum-filtered and thoroughly washed with deionized water. It was then subjected to acid pre-extraction with HCl-adjusted water (pH 4.0) in a water bath at 65 °C under constant agitation at 400 rpm for 3 h. This step facilitated the release of calcium ions and other cations, converting alginate salts into alginic acid prior to alkaline extraction.

Next, the pretreated sample was placed in a beaker containing 40 parts deionized water per part of dry seaweed, carefully adjusted to pH 10 with Na_2_CO_3_ (Merck, PHR1948), and gently stirred at 70 °C for 3 h, ensuring the complete conversion of alginic acid into sodium alginate and the controlled release of hydrogen ions. These heating stages at 65 and 70 °C were of limited duration and were selected within the range commonly reported for alginate extraction workflows; accordingly, the concern discussed above refers to prolonged and/or harsh thermal-chemical exposure, rather than to isolated processing steps conducted at or slightly above 60 °C under controlled conditions.

The resulting paste was vacuum-filtered using a fine nylon filter cloth (100 µm) and gently squeezed to extract the liquid. The filtered solution was heated to 55 °C, and a 10% CaCl_2_ solution (1.0 meq/g dry algae) (Merck, C4901), was slowly added under continuous agitation to form solid calcium alginate efficiently. The precipitate was recovered by centrifugation at 5000 rpm (~4000× *g*) for 15 min at 4 °C. The precipitated calcium alginate was carefully resuspended in deionized water at a 1:30 (*w*/*v*) ratio, then adjusted to pH 1.5 with HCl (Merck, 1003171000) under continuous monitoring to obtain pure fibrous alginic acid. This procedure was repeated twice to ensure maximum purity and minimize residual impurities (Figure 1A).

Finally, the alginic acid obtained was mixed with a 60% hydroalcoholic ethanol solution, and the pH was carefully adjusted to 8.0 using a fresh 10% Na_2_CO_3_ solution (Merck, 451614), while maintaining constant agitation at 500 rpm for 2 h. This step converted alginic acid completely into highly pure sodium alginate. The resulting sodium alginate was washed three times with cold absolute ethanol to remove residual salts and impurities, then carefully dried at 45 °C for 24 h in a forced-convection oven. A detailed, updated schematic of the optimized procedure for obtaining alginate is shown in Figure 1B,C.

### 2.2. Calculation of Alginate Extraction Yield

To determine the extraction yield, Equation (1) was solved:

(1) $$\%R=\frac{m_{f}}{m_{i}}\cdot 100$$

where %R is the extraction yield, $m_{i}\;$ is the initial dry algae mass $m_{f}$ is the mass of sodium alginate obtained.

The extraction procedure was performed in triplicate (*n* = 3), and the extraction yield is reported as mean ± standard deviation (SD).

### 2.3. UV–Vis Spectroscopy

UV–Vis absorption spectra (190–350 nm) were recorded using a UV–Vis spectrophotometer (Thermo Scientific, Evolution One Plus UV-Vis spectrophotometer, Waltham, MA, USA). Two types of alginate solutions were analyzed: (1) sodium alginate extracted from *S. fluitans*, and (2) a commercial sodium alginate standard (Merck, W201502), each prepared at a concentration of 100 μg/mL. Deionized water was used as a blank reference. Spectral measurements were performed at room temperature using quartz cuvettes with a 1 cm path length.

### 2.4. Fourier Transform Infrared Spectroscopy (FTIR)

FTIR spectra of sodium alginate extracted from *S. fluitans* and a commercial sodium alginate standard (Merck, W201502) were collected using a Spectrum Two^TM^ Fourier transform infrared spectrometer (PerkinElmer Inc., Waltham, MA, USA). Samples were prepared as KBr pellets, and spectra were recorded over the 4000–400 cm^−1^ range at a spectral resolution of 4 cm^−1^ with 32 accumulated scans.

### 2.5. Nuclear Magnetic Resonance (NMR) Spectroscopy

#### 2.5.1. ^1^H NMR Spectroscopy

^1^H NMR spectra were recorded on a Bruker Avance III HD spectrometer (Bruker BioSpin, Germany) operating at 500 MHz. Sodium alginate extracted from *S. fluitans* (approximately 3.5 mg) was dissolved in 600 µL of D_2_O containing 0.05 wt% DSS as an internal reference. Samples were gently heated to ensure complete dissolution and analyzed at 25 °C without sample spinning using the standard zg pulse sequence. Acquisition parameters were as follows: 64 scans, acquisition time of 4 s, relaxation delay of 2 s, spectral width of 20 ppm, and a 90° pulse angle. Chemical shifts (δ) were referenced to DSS at 0.0 ppm. Spectra were processed using MestReNova software v14.2.3-2924, and peak assignment and compositional analysis were performed according to standard alginate ^1^H NMR procedures and ASTM F2259-10 (2012) [18]. FALTA AGREGAR LA SIGUIENTE CITA CON SU GESTOR POR FAVOR, F2259-10; Standard Test Method for Determination of the Composition of Alginates by Proton Nuclear Magnetic Resonance (1H NMR) Spectroscopy. ASTM International: West Conshohocken, PA, USA, 2012.

#### 2.5.2. ^13^C NMR Spectroscopy

^13^C NMR spectra were acquired on a JEOL ECZ 600R spectrometer (JEOL Ltd., Tokyo, Japan) operating at 150 MHz for ^13^C detection. Sodium alginate extracted from *S. fluitans* (20 mg) was dissolved in 1 mL of D_2_O and heated to 80 °C under gentle stirring to ensure complete dissolution. Approximately 600 µL of the resulting viscous solution were transferred into 5 mm NMR tubes. Spectra were recorded at 80 °C using a single-pulse decoupled gated NOE experiment (pulse sequence: carbon.jxp). Acquisition parameters included 18,975 scans, an acquisition time of 0.7 s, a TD of 33,687, a relaxation delay of 2.28 s, a spectral width of 250 ppm, and a 90° pulse angle. Chemical shifts (δ) were referenced to DSS at 0.0 ppm. Spectra were processed with MestReNova software v14.2.3-2924, and peak assignment was based on characteristic chemical shifts reported for alginate in the literature.

### 2.6. Synthesis of Nanoparticles

Alginate nanoparticles were synthesized from Sargassum-derived sodium alginate by a modified ionotropic gelation method. Briefly, 0.25 g of sodium alginate was dissolved in 99 mL of deionized water containing 1 mL of natural honey, which acted as a natural stabilizing agent to improve colloidal stability and reduce nanoparticle aggregation during synthesis [19], under magnetic stirring at 500 rpm until a homogeneous solution was obtained. Subsequently, 0.450 mL of a 1% CaCl_2_ solution was added dropwise to promote ionic crosslinking, and the mixture was stirred continuously at 700 rpm for 4 h. The resulting suspension was centrifuged at 3500 rpm for 5 min and sequentially vacuum-filtered through 0.45 μm membranes to remove larger aggregates prior to physicochemical characterization.

### 2.7. Scanning Electron Microscopy (SEM)

For SEM analysis, an aliquot of the nanoparticle suspension was deposited onto aluminum stubs, dried under ambient conditions, sputter-coated with a thin carbon layer, and examined using a JEOL JSM-5600LV scanning electron microscope operated at 20 kV. Micrographs were acquired at various magnifications to characterize the sample surface thoroughly.

### 2.8. Dynamic Light Scattering (DLS) and Colloidal Stability

Dynamic light scattering (DLS) measurements were performed to evaluate the colloidal stability of the alginate nanoparticle dispersions. DLS was used to determine the hydrodynamic diameter and polydispersity index (PDI) of the particles in suspension based on fluctuations in scattered light generated by Brownian motion. Measurements were carried out using a Zetasizer Pro (Malvern Panalytical, Malvern, UK). The dispersions were analyzed at 0, 24, and 72 h after preparation, and samples were maintained at room temperature throughout the study. Colloidal stability was assessed from changes in hydrodynamic diameter and PDI over time.

## 3. Results and Discussion

### 3.1. Yield of Alginate

The extraction yield (%R) of sodium alginate from *S. fluitans*, calculated using Equation (1), was 18.7 ± 0.05% (mean ± SD, *n* = 3), which falls within the range reported for comparable acid/base extraction methods [16,17]. Because the biochemical composition of the starting biomass was not independently determined, this value should be interpreted as extraction yield relative to dry feedstock, not as a direct estimate of the total alginate content originally present in the raw material. This demonstrates that the employed controlled extraction conditions enabled alginate recovery while limiting excessive polymer degradation. Although purity was not determined as an independent quantitative percentage, the extracted material yielded precise and interpretable NMR spectra, and UV–Vis, FTIR, and NMR analyses supported its assignment as alginate. Importantly, the aim of this study was not to claim a fundamentally novel low-temperature or reduced-chemical extraction route, but to show that alginate recovered from pelagic *S. fluitans* under a literature-based protocol retained structural features compatible with direct nanoparticle synthesis. In this context, the discussion of thermal effects has been nuanced accordingly: degradation risk is associated with prolonged and/or harsh thermal–chemical exposure, whereas the present workflow used discrete heating stages of limited duration.

### 3.2. Characterization of Alginate Extracted from S. fluitans

#### 3.2.1. UV–Vis Spectral Analysis of Sargassum-Derived Alginate

**Figure 2:**
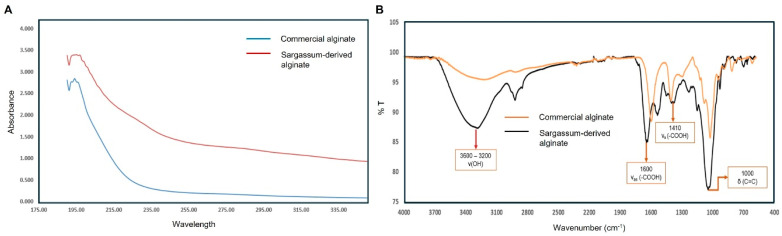
UV–Vis and FTIR characterization of commercial alginate and Sargassum-derived alginate. (**A**) UV–Vis absorbance spectra recorded in the 175–350 nm range. (**B**) FTIR transmittance spectra (4000–400 cm^−1^) showing the characteristic bands of alginate. The overall similarity between the spectra of the extracted sample and the commercial standard supports the successful extraction of alginate from *Sargassum*.

The UV–Vis spectra of Sargassum-derived and commercial alginate are shown in Figure 2A. Both samples exhibited the characteristic strong absorption of alginate below 200 nm, with a maximum around 195–197 nm associated with hydroxyl and carboxyl groups, followed by a progressive decrease at higher wavelengths, as expected for alginate solutions [20]. However, the Sargassum-derived alginate showed markedly higher absorbance throughout 190–350 nm than the commercial sample, suggesting a higher content of chromophoric groups and/or residual impurities. This pattern is consistent with previous reports indicating that crude alginate from Sargassum may contain co-extracted compounds that generate additional absorbance in the 230–280 nm region, including polyphenols, proteins, flavonoids, and nucleic acids [21,22]. Thus, although the overall spectral profile confirms successful alginate extraction, the higher absorbance relative to commercial alginate indicates that further purification would likely improve its quality and produce a spectral profile closer to commercial standards [23].

#### 3.2.2. FTIR Spectrum of Sodium Alginate from *S. fluitans*

Figure 2B presents the FTIR spectrum of alginate extracted from *S. fluitans*, characterized by several prominent absorption bands typical of alginate biopolymers. The broad and intense absorption band between 3500 and 3000 cm^−1^ primarily corresponds to the O–H stretching vibrations of hydroxyl groups, reflecting the polysaccharide’s extensive hydrogen bonding network [24,25]. The absorption at approximately 2900 cm^−1^ is attributed to the asymmetric and symmetric stretching vibrations of aliphatic C–H groups, characteristic of the alginate polymer backbone [25,26].

The FTIR spectrum of sodium alginate extracted from *S. fluitans* displays all the characteristic bands expected for an alginate polysaccharide and closely matches literature reports for alginates from brown seaweeds [6,24]. A broad, strong absorption in the 3500–3200 cm^−1^ region (centered around ~3400 cm^−1^) is observed, which corresponds to O–H stretching vibrations of hydroxyl groups and inter-molecular hydrogen-bonded water in the alginate structure. A much weaker peak near 2920 cm^−1^ is attributed to C–H stretching of aliphatic CH bonds in the uronate sugar rings [26]. The most prominent bands appear in the fingerprint region: a dominant peak around 1600 cm^−1^ and a shoulder/second peak near 1400 cm^−1^. These two bands are assigned to the asymmetric and symmetric stretching vibrations of the carboxylate (–COO^−^) groups present on the alginate’s mannuronic and guluronic acid units [6,26]. In particular, the strong band in the 1590–1630 cm^−1^ range corresponds to the –COO− asymmetric stretch, while the one near 1400–1420 cm^−1^ is due to the –COO− symmetric stretch (the latter being weaker) [25,26]. These carboxylate peaks confirm the presence of alginate’s uronic acid residues, as they are a signature of the polymer’s carboxylate functionality. Only a very faint signal around ~1720 cm^−1^, if present, would be compatible with residual protonated carboxylic acid (C=O) groups in sodium alginate [25].

In the lower-wavenumber region, the alginate spectrum exhibits a series of bands that correlate with its sugar-ring skeleton. Strong absorptions around 1100–1020 cm^−1^ are attributed to C–O stretching vibrations of the polysaccharide backbone (C–O–C linkages and secondary alcohol C–O groups in the uronate units) [24,26]. Additionally, characteristic smaller peaks appear in the 950–800 cm^−1^ region (the “anomeric” region for carbohydrates). These have been linked to ring vibrations and C–H deformation modes of the alginate’s monomers: signals around 930–950 cm^−1^ are associated with C–O stretching of uronic acid residues, while bands near 890–870 cm^−1^ are attributed to C1–H deformation of β-mannuronic acid units [24,25]. Minor peaks in the 820–850 cm^−1^ range have also been assigned to vibrations of mannuronic vs. guluronic residues in the alginate chain [25]. Overall, the FTIR profile of the extracted sample, from the broad O–H band to the carboxylate and C–O bands, is in excellent agreement with reported spectra of sodium alginate [26]. This correspondence with the peer-reviewed literature confirms that the acid/base extraction yielded a typical sodium alginate structure from *S. fluitans*, characterized by abundant hydroxyl and carboxylate functional groups and a glycosidic polysaccharide backbone, as evidenced by the FTIR bands identified.

### 3.3. NMR Analysis

#### 3.3.1. ^1^H NMR Analysis and Alginate Composition

**Figure 3:**
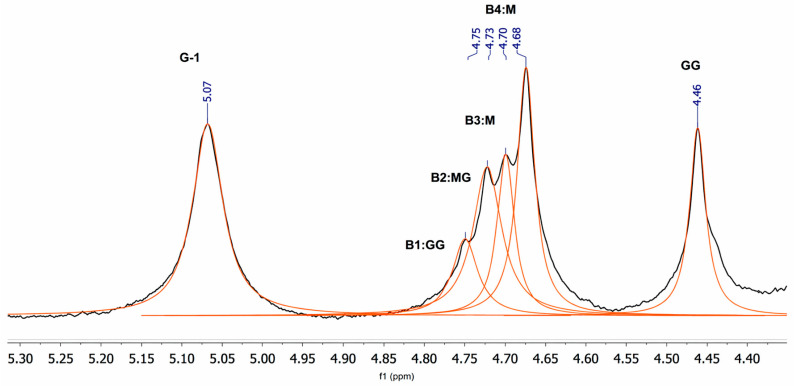
Expanded low-field region of the ^1^H NMR spectrum of alginate isolated from *Sargassum* biomass, showing peak deconvolution and assignment of the resolved signals. The experimental spectrum is shown as the black trace, whereas the fitted individual components are shown in orange. Six signals were resolved at δ 5.07 (G-1), 4.75 (B1:GG), 4.72 (B2:MG), 4.70 (B3:M), 4.67 (B4:M), and 4.46 ppm (GG-5). Quantitative integration revealed that the B-region (B1–B4, δ 4.75–4.67 ppm) accounted for 67.93% of the total integrated area, with B2:MG the most intense component (19.09%) and GG-5 the least intense (13.20%). The complete integration data are presented in Table 1.

The ^1^H NMR spectrum of the extracted alginate (500 MHz, D_2_O, 25 °C) showed the characteristic low-field signals of alginate, including a well-defined signal at δ 5.07 ppm assigned to the anomeric proton of α-L-guluronate (G-1), a cluster of sequence-sensitive signals between δ 4.75 and 4.67 ppm (B1–B4), and a distinct signal at δ 4.46 ppm assigned to the GG-5 signal [27]. The B1–B4 region contains overlapping sequence-dependent signals conventionally assigned to G-5 in GGM and MGM environments (B1 and B2) and to M-1 in MG and MM environments (B3 and B4). The broader proton envelope outside this diagnostic low-field region should be interpreted with caution because extensive signal overlap is common in alginate ^1^H NMR spectra acquired in D_2_O, whereas compositional calculations are typically based on the diagnostic low-field signals assigned by Grasdalen and co-workers [28,29]. This interpretation is consistent with the classical alginate NMR framework established by Grasdalen and co-workers and later applied to *Sargassum*-derived alginates (Figure 3) [28,29,30]. Throughout this section, δ values are reported as chemical shifts, whereas the observed spectral features are referred to as signals.

The low-field region of the alginate ^1^H NMR spectrum was deconvoluted into six resolved components, and the corresponding chemical shifts, integrated areas, and relative contributions are summarized in Table 1. A total integrated area of 3833.96 arbitrary units was obtained for this spectral window. The distribution of signal intensities was heterogeneous, with the B2:MG signal at δ 4.72 ppm showing the highest contribution (731.99 a.u., 19.09% of the total area), followed by the signal assigned to G-1 at δ 5.07 ppm (723.58 a.u., 18.87%) and the signal assigned to B4:M at δ 4.67 ppm (716.44 a.u., 18.69%). In contrast, the GG-5 signal at δ 4.46 ppm exhibited the lowest relative contribution (505.93 a.u., 13.20%). Notably, the central overlapped region comprising peaks B1-B4 (δ 4.75–4.67 ppm) accounted for 67.93% of the total integrated area, confirming that this cluster dominates the spectral profile. The order of relative abundance was B2:MG > G-1 > B4:M > B1:GG > B3:M > GG-5, indicating a heterogeneous distribution of sequence-sensitive environments in the alginate isolated from *Sargassum* biomass. Because these values derive from deconvoluted NMR signals, they should be interpreted as relative spectral contributions within the analyzed region; conversion into absolute block composition requires the corresponding microstructural calculation model [31].

Importantly, the preservation of short GG domains is structurally relevant because contiguous guluronate residues constitute the preferred binding motifs for Ca^2+^-mediated junction-zone formation in the classical egg-box model. Accordingly, although the extracted alginate is M-rich, its ^1^H NMR profile still supports its ability to undergo ionotropic crosslinking, which is consistent with its subsequent use in Ca^2+^-crosslinked nanoparticle formulations [31].

The compositional and sequence parameters calculated from the diagnostic ^1^H NMR signals are summarized in Table 2. The extracted alginate exhibited a predominance of mannuronate residues (F^M^ = 0.61) over guluronate residues (F^G^ = 0.39), corresponding to an M/G ratio of 1.54. The calculated number-average length of guluronate blocks longer than one residue (N^G>1^ = 2.42) indicates that guluronate residues are mainly arranged in short consecutive sequences rather than in extended G-rich domains. Overall, these data are consistent with an M-rich alginate containing short GG blocks within a mixed blockwise structure.

#### 3.3.2. ^13^C NMR Analysis

**Figure 4:**
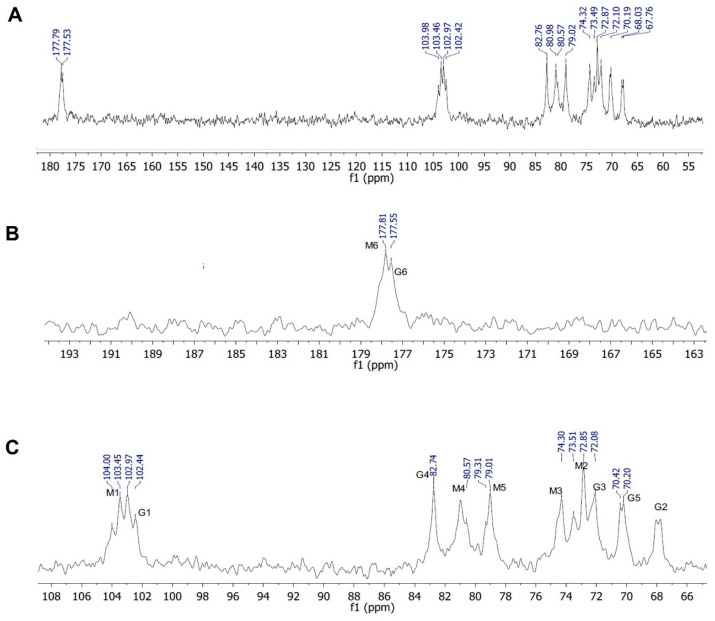
^13^C NMR spectra of *Sargassum*-derived alginate recorded in D_2_O (600 MHz, 80 °C). (**A**) Full ^13^C NMR spectrum showing the characteristic signals of alginate. (**B**) Expanded view of the carboxyl region, with signals at δ 177.79 and 177.53 ppm assigned to the C6 carbons of mannuronic (M6) and guluronic (G6) residues, respectively. (**C**) Expanded view of the anomeric and ring-carbon region, showing the characteristic signals of both mannuronic and guluronic units, namely, M1/G1, G4, M4, M5, M2/M3, G3, G5, and G2. The overall spectral pattern is consistent with the typical ^13^C NMR profile of alginate and confirms the presence of both β-D-mannuronic acid and α-L-guluronic acid residues in the extracted polymer [32,34].

Figure 4 shows the ^13^C NMR spectrum of the alginate extracted from *Sargassum*. The spectrum displayed the diagnostic carbon signals of alginate, confirming the presence of both β-D-mannuronic acid (M) and α-L-guluronic acid (G) residues. Two resolved signals in the carboxylate region at δ 177.81 and 177.53 ppm were assigned to M6 and G6, respectively. In the anomeric region, a multiplet extending from δ 104.00 to 102.42 ppm was observed and attributed to sequence-sensitive M1 and G1 carbons. Additional signals were detected at δ 82.76 ppm (G4), 80.98–79.01 ppm (M4/M5), 74.30–72.08 ppm (overlapping M2/M3 signals), 70.42–70.20 ppm (G3/G5), and near δ 68.0 ppm (G2). These chemical shifts are characteristic of alginate and agree well with the seminal ^13^C NMR assignments reported for alginate, in which the chemical shifts in the carbon atoms were shown to be strongly influenced by the local M/G sequence [30,32]. The multiplicity observed in the anomeric and ring-carbon regions is therefore consistent with a sequence-heterogeneous copolymer rather than a uniform repeating structure [28,30,33]. This interpretation is consistent with the classical description of alginate as a block copolymer comprising MM, GG, and MG domains along the chain. Although monomer composition was determined independently from the ^1^H NMR data, the present ^13^C spectrum provides clear structural confirmation that the extracted polymer corresponds to alginate with the mixed M/G architecture expected for brown-algal polysaccharides. The absence of major additional signals further suggests that the isolated material was enriched in alginate. Similar NMR-based structural behavior has previously been reported for alginates isolated from *Sargassum* spp. [30].

### 3.4. Comparison with Other Brown Algal Alginates

The obtained alginate showed an M/G ratio of ~1.54, corresponding to ~61% M and 39% G, which places it at the upper end of the range typically reported for *Sargassum* alginates and indicates a more mannuronate-rich composition than is usually described for *S. fluitans*. Most studies on pelagic *Sargassum*, particularly *S. fluitans*, report lower M/G ratios and therefore more G-rich alginates. For example, *S. fluitans* from the West Indies has been reported to exhibit M/G values of ~0.59 and ~0.50–0.51, while even more G-rich fractions have been described, with M/G as low as ~0.15 in frond-derived material. By contrast, other warm-water *Sargassum* species such as *S. siliquosum* may reach M/G values of ~0.7–0.9. Overall, the literature suggests that *Sargassum* alginates generally fall within M/G ≈ 0.8–1.5 depending on species, tissue source, and environmental conditions [7,34,35,36]. Thus, although the composition of our sample is unusual for *S. fluitans*, it remains within the broader variability reported for brown algal alginates and may reflect local growth conditions or the specific thallus region analyzed.

The relatively short average guluronate block length (N^G>1^ ≈ 2.42) is consistent with this moderate G content, indicating that G residues are present but do not form long contiguous G-blocks. This contrasts with highly G-rich alginates, such as those from *Laminaria hyperborea* stipes, which contain ~70% G (M/G~0.4) and very long G-blocks (N^G>1^~15), features associated with the formation of firm, brittle calcium-crosslinked gels [37]. By comparison, the composition of the sample closely resembles that of *Macrocystis pyrifera* alginate, for which ~61% M and 39% G (M/G~1.56) have been reported. Such M-rich alginates generally form softer, more elastic gels, whereas G-rich alginates yield stronger but more fragile networks. In general, high M/G ratios (>1), together with a predominance of M-rich and alternating sequences, favor flexible, compliant hydrogels. In contrast, low M/G ratios (<1) and abundant G-blocks promote stiffer, more brittle materials [7,27,38]. Accordingly, the intermediate composition of this sample suggests gelation behavior that is more elastic than that of G-dominated *Sargassum* alginates, while still retaining sufficient G content to support effective ionic crosslinking. From a nanoparticle formulation perspective, this also suggests that under otherwise similar ionotropic gelation conditions, the present alginate would be expected to generate less compact Ca^2+^-crosslinked matrices than more G-rich alginates, which is directly relevant when interpreting particle size and short-term colloidal stability.

The ^1^H NMR data further support a mixed-block microstructure. The presence of the GG-5 signal confirms that GG diads are present, and therefore, that some GGG triads and short G-blocks likely occur, although not at high frequency. At the same time, the signals assigned to M-1 and the corresponding M–M splitting patterns indicate the presence of MM diads, while the resolved sub-peaks in the mannuronate region suggest a substantial contribution of alternating MG/GM sequences. This interpretation is consistent with the calculated block lengths and places the sample between the two extremes described in the literature: highly blocky, G-rich alginates such as frond alginate from *S. fluitans*, and more intermixed structures such as those reported for *S. siliquosum* [27]. Overall, the sequence pattern of this alginate appears balanced, with moderate blockiness and a substantial proportion of alternating segments. Such structures are often desirable because they combine gel strength with elasticity, suggesting that this alginate may be particularly suitable for applications requiring resilient, non-brittle hydrogels.

### 3.5. Tyndall Effect and DLS Analyses

**Figure 5:**
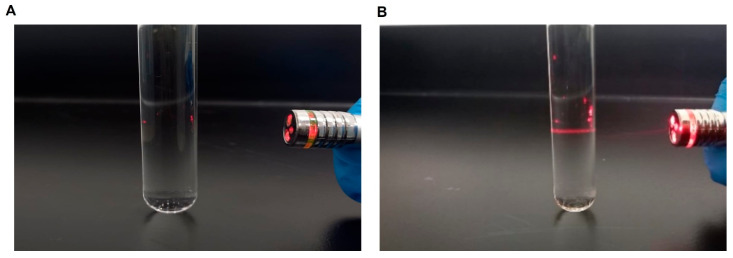
Tyndall effect photographs of the blank control and alginate nanoparticle dispersion under red-laser illumination. (**A**) Blank control. (**B**) Alginate nanoparticle dispersion. The more clearly visible laser path in panel B is consistent with light scattering by colloidal particles.

Following extraction, the sodium alginate obtained from pelagic *S. fluitans* was used as the polymeric precursor for nanoparticle fabrication by ionotropic gelation, as previously described. Specifically, the formulation consisted of alginate crosslinked with Ca^2+^ in the presence of honey as a stabilizing agent; accordingly, the resulting dispersion is referred to here as honey-stabilized calcium alginate nanoparticles. To obtain rapid qualitative evidence of colloidal formation, the Tyndall effect was evaluated by illuminating the dispersions with a red laser (Figure 5). The blank control (A) showed only weak reflections from the vial walls and no clearly continuous beam, whereas the alginate nanoparticle dispersion (B) displayed a distinct visible light path through the sample. This behavior is consistent with light scattering by colloidal particles, and therefore, supports the formation of a nanoparticulate dispersion. Because the Tyndall effect is a qualitative test, it should be interpreted as complementary evidence and not as a stand-alone demonstration of particle size or morphology.

**Figure 6:**
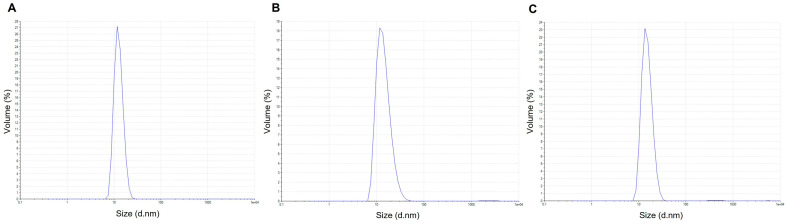
Volume-based hydrodynamic size distribution of alginate nanoparticles determined by dynamic light scattering (DLS) at room temperature. Measurements were performed at (**A**) 0 h, (**B**) 24 h, and (**C**) 72 h after preparation using a Zetasizer Pro, particle size analyzer (Malvern Panalytical Ltd., Malvern, UK). The distributions were used to evaluate the colloidal stability of the nanoparticle dispersion over time (*n* = 3).

DLS was used to evaluate the short-term colloidal behavior of the alginate nanoparticle suspension after 72 h of storage at room temperature. The volume-based size distributions remained dominated by a single nanoscale population throughout the storage period. At 0 h (Figure 6A), the main peak was centered at 12.75 nm and accounted for 99.9% of the total volume, with a Z-average of 37.7 nm and a PDI of 0.291. After 24 h (Figure 6B), the principal population was centered at 14.60 nm (99.8%), with a Z-average of 19.98 nm and a PDI of 0.229. At 72 h (Figure 6C), the dominant peak was observed at 15.31 nm (99.7%), while the Z-average increased to 70.66 nm and the PDI was 0.286. Minor secondary populations in the submicrometric-to-micrometric range were detected at very low abundance (≤0.2%), indicating only trace aggregation during storage. Overall, the slight shift in the main peak position and the relatively small variation in PDI suggest that the nanoparticle dispersion remained reasonably stable over 72 h under ambient conditions.

The present DLS results place this formulation at the small-size end of the range reported for alginate-based nanoparticles. Honey-stabilized calcium alginate nanoparticles produced by ionic crosslinking have previously been reported in the 10–100 nm range, whereas other ionotropically gelled alginate nanoparticle systems commonly exhibit mean sizes in the ~108–242 nm or ~135–228 nm range, depending on alginate molecular characteristics, including monomer composition and M/G-dependent block structure, polymer/crosslinker concentration, and homogenization conditions [19,39,40]. This compositional parameter is pertinent in the present case because the extracted alginate was M-rich (M/G = 1.54), closely resembling the M/G value reported for *Macrocystis pyrifera* alginate (~1.56) and differing from the more G-rich *Sargassum* alginates often described in the literature [7,27,34,35,36]. Under otherwise similar ionotropic gelation conditions, such an M-rich profile is expected to generate less densely Ca^2+^-crosslinked structures. In this context, the predominance of a small hydrodynamic population and the limited variation in PDI over 72 h are consistent with the behavior expected for an M-rich alginate nanoparticle system. The PDI values obtained here (0.229–0.291) are also comparable to those reported for relatively uniform alginate nanoparticle dispersions. Because the M/G ratio is not systematically reported in many published nanoparticle studies, strict one-to-one comparison remains limited; nevertheless, the present results are consistent with mild ionotropic gelation of an M-rich alginate matrix. Taken together, these data support the persistence of a dominant nanoscale alginate population during short-term storage at room temperature, although the occasional larger peaks suggest that limited aggregate formation may occur over time.

### 3.6. SEM Morphology of Honey-Stabilized Alginate Nanoparticles

**Figure 7:**
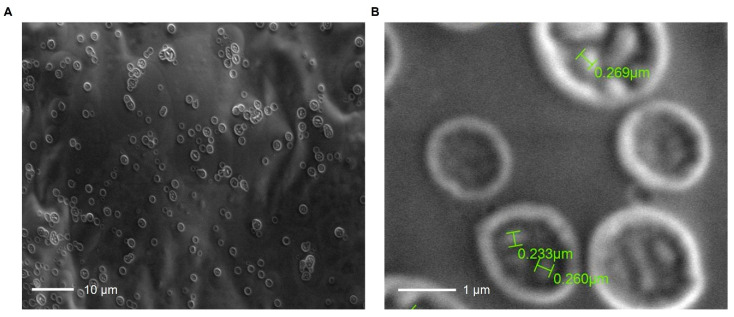
Scanning electron microscopy (SEM) images of honey-stabilized alginate nanoparticles prepared from Sargassum-derived alginate. (**A**) Low-magnification micrograph showing the overall distribution of the nanoparticles on the sample surface. (**B**) Higher-magnification image revealing their predominantly spherical-to-subspherical morphology, with representative dry particle diameters ranging from 0.233–0.269 µm. Scale bars: 10 µm in (**A**) and 1 µm in (**B**).

Then, SEM micrographs revealed numerous discrete, predominantly sub-spherical particles with limited local aggregation (Figure 7). Representative dry diameters measured from the higher-magnification image ranged from 233 to 269 nm. These values should be interpreted as indicative dry particle sizes rather than as a complete particle size distribution. At higher magnification, representative dry diameters ranged from 233 to 269 nm, confirming the formation of sub-300 nm calcium-crosslinked alginate nanoparticles. This dimensional interval is consistent with ionically gelled alginate systems, in which alginate concentration, CaCl_2_ concentration, homogenization or stirring conditions, and the polymer’s intrinsic M/G-dependent crosslinking behavior can all influence particle size and dispersity [41]. Comparable alginate nanoparticles have been reported as spherical and uniformly distributed in the 134.9–228.0 nm range [40]. At the same time, honey-loaded alginate nanoparticles produced via ionic crosslinking have also exhibited a spherical morphology, with an average size of 312 ± 4.32 nm [42]. In this context, the present dry particle size range (233–269 nm) lies between those reports and is compatible with a formulation prepared from an M-rich alginate precursor (M/G = 1.54), which would be expected to form a somewhat less compact calcium-alginate network than highly G-rich systems under similar crosslinking conditions.

The bright peripheral rims and darker central regions observed in many particles should be interpreted cautiously. For hydrated polysaccharide nanoparticles, conventional high-vacuum SEM requires dehydration and a conductive coating, both of which can cause the native hydrogel network to condense, shrink, or partially collapse, thereby modifying the apparent morphology [43]. Therefore, the annular contrast observed here is more likely related to drying-induced topographic effects than to unequivocal hollow-core formation. This interpretation is consistent with previous reports on honey-stabilized calcium alginate nanoparticles, where slight aggregation became evident after drying [19].

Honey may also contribute to the final microstructure of the formulation. In alginate-based systems, honey has been reported to alter surface morphology, reduce drying-associated shrinkage and crack formation, and modify the organization of the polymer network in a concentration-dependent manner [19,44].

## 4. Conclusions

This study demonstrates a feasible route for converting pelagic *Sargassum* biomass into value-added alginate nanomaterials. Under controlled multistep extraction conditions, the recovered sodium alginate retained the characteristic structural features of the native polymer, was recovered with an extraction yield of 18.7 ± 0.05% (mean ± SD, *n* = 3) relative to dry feedstock, and exhibited an M-rich composition compatible with ionotropic crosslinking. The extraction procedure used here should be interpreted as a literature-based protocol applied to pelagic *S. fluitans*, rather than as a claim of fundamentally new extraction chemistry. The resulting nanoparticle formulation predominantly yielded sub-spherical particles, with representative dry diameters of 233–269 nm, as measured by SEM. In addition, DLS measurements showed a dominant population of nanoscale particles over 72 h at room temperature, with the main volume-based peaks between 12.75 and 15.31 nm and PDI values ranging from 0.229 to 0.291, indicating reasonable short-term colloidal stability. Accordingly, the principal novelty of this work lies in demonstrating that non-commercial alginate extracted from pelagic *S. fluitans* can be structurally verified and then used directly to produce calcium-crosslinked alginate nanoparticles. Rather than treating *Sargassum* solely as a coastal nuisance, these findings support its use as a marine feedstock for structurally verified alginate and colloidally stable alginate-based nanoparticles with potential relevance to biomedical and related biotechnological applications, subject to controlled biomass collection, prompt preprocessing, and application-specific quality and safety evaluation. Because the biochemical composition of the starting biomass was not independently determined, the reported extraction yield should not be interpreted as a direct estimate of the total alginate content originally present in the raw material. Although further studies are needed to assess application-specific performance, long-term storage stability, and safety, the present findings provide a solid foundation for transforming recurrent *Sargassum* influxes into higher-value materials.

## Figures and Tables

**Table 1:** Chemical shifts, integrated peak areas, and relative contributions of the deconvoluted ^1^H NMR signals of alginate isolated from *Sargassum* biomass.

Peak	δ (Chemical Shift, ppm)	Area	% of Total
G-1	5.07	723.58	18.87
B1:GG	4.75	593.79	15.49
B2:MG	4.72	731.99	19.09
B3:M	4.7	562.23	14.66
B4:M	4.67	716.44	18.69
GG-5	4.46	505.93	13.2

**Table 2:** Compositional and sequence parameters of the extracted alginate determined from diagnostic ^1^H NMR signals.

Parameter	Value
F^M^ (mannuronate fraction)	0.61
F^G^ (guluronate fraction)	0.39
M/G ratio	1.54
N^G>1^ (avg. G-block length)	2.42

F^M^, mannuronate fraction; F^G^, guluronate fraction; M/G ratio, mannuronate-to-guluronate ratio; N^G>1^, number-average length of guluronate blocks longer than one residue.

## Data Availability

The raw data supporting the conclusions of this article will be made available by the authors on request.
